# Association between maternal smoking during pregnancy and developmental disabilities in US children and adolescents: A cross-sectional study from NHANES

**DOI:** 10.18332/tid/200339

**Published:** 2025-02-10

**Authors:** Haiyan Miao, Canfei Zhang, Jing Qian, Hong Jing, Hui Nan, Shasha Li, Xiahui Shen, Jinxia Zhao

**Affiliations:** 1 Department of Children's Health Care, People's Hospital of Liaocheng, Liaocheng, People’s Republic of China; 2 Department of Pharmacology, People's Hospital of Liaocheng, Liaocheng, People’s Republic of China

**Keywords:** cross-sectional study, smoking during pregnancy, developmental disabilities, National Health and Nutrition Examination Survey

## Abstract

**INTRODUCTION:**

Maternal smoking during pregnancy is associated with placental DNA methylation and RNA expression, offspring DNA methylation, and affects the decline of mature neurons and the prenatal human brain development trajectory.

**METHODS:**

This study is a secondary analysis of data from the National Health and Nutrition Examination Survey (NHANES) spanning 2003 to 2008, comprising 10111 children and adolescents. Inclusion criteria required participants to have complete questionnaire responses regarding maternal smoking during pregnancy and receipt of special education or early intervention services. The risk of developmental disabilities was assessed using a multifactor logistic regression model.

**RESULTS:**

In the cohort of 10111 children and adolescents, 727 (7.2%) received special education or early intervention services. Of these participants, 1504 (14.9%) were exposed to maternal smoking during pregnancy. The prevalence of maternal smoking was higher (12.3%) in the group receiving special education or early intervention compared to those who did not (6.3%). After adjusting for other relevant factors in a multifactorial logistic regression model, maternal smoking during pregnancy was significantly associated with an increased likelihood of requiring special education or early intervention services (adjusted odds ratio, AOR=1.51; 95% CI: 1.24–1.83, p<0.001).

**CONCLUSIONS:**

This cross-sectional analysis found an association between maternal smoking during pregnancy and the need for special education or early intervention services among US children and adolescents, after adjusting for confounding variables. Our findings suggest that maternal smoking during pregnancy may increase the odds of developmental disabilities.

## INTRODUCTION

The Individuals with Disabilities Education Act (IDEA) in the United States mandates free and appropriate education for students with disabilities^1^. Part B of IDEA focuses on preschool and special education programs for school-aged children, while Part C is dedicated to newborns and toddlers aged 0–36 months, requiring the provision of an Early Intervention Program (EIP)^1^. The scope of special education services primarily includes disabilities related to learning, speech, or language, but also encompasses a variety of health disorders such as mental health issues, intellectual disabilities, developmental delays, and autism^2^. Additionally, multiple disabilities, hearing impairments, and orthopedic injuries, though less common, are covered^2^.

Recent years have witnessed a notable increase in the prevalence of developmental disabilities in the US, increasing from 16.22% in 2009–2011 to 17.76% in 2015–2017^3^. These conditions significantly impact individuals and their families, potentially leading to lower educational achievement, diminished quality of life, and increased healthcare costs^4,5^. Among the myriad factors contributing to developmental disabilities, *in utero* exposure is critically significant^6^. Prenatal neurodevelopment plays a crucial role in the emergence of neurological disorders later in life, given the vulnerability of the fetus to the maternal environment^6^.

The association between maternal smoking during pregnancy and various risks is well-established, yet maternal smoking remains prevalent. Data from 2010 to 2017 indicate that 8.1% of mothers who gave birth reported smoking during pregnancy^7^. Smoking during this critical period poses dangers not only to the mother’s health but also to the developing fetus^8-10^, contributing to developmental disabilities as highlighted by extensive research^11-13^. Despite this evidence, the direct causality of this association remains a topic of debate^14-16^.

It is hypothesized that children and adolescents exposed to maternal smoking during pregnancy are more likely to experience developmental disabilities, necessitating services such as special education or early intervention programs, compared to those with no exposure. To explore this hypothesis, an analysis was conducted using a representative sample of US children aged 1–15 years, drawing data from the National Health and Nutrition Examination Survey (NHANES). The objective was to examine the association between maternal smoking during pregnancy and the odds of requiring special education or early intervention services.

## METHODS

### Study participants

This study is a secondary analysis of a dataset from the NHANES 2003–2008, conducted by the US Centers for Disease Control and Prevention^17,18^. These years were selected based on the availability of the most recent NHANES data at the time of the study. NHANES aims to assess the health and nutritional status of the non-institutionalized US population through a stratified multistage probability sampling method. The data collection was overseen by the National Center for Health Statistics of the US and was conducted following the approval of its ethics review board^19^, and participants provided written informed consent.

**Figure 1:**
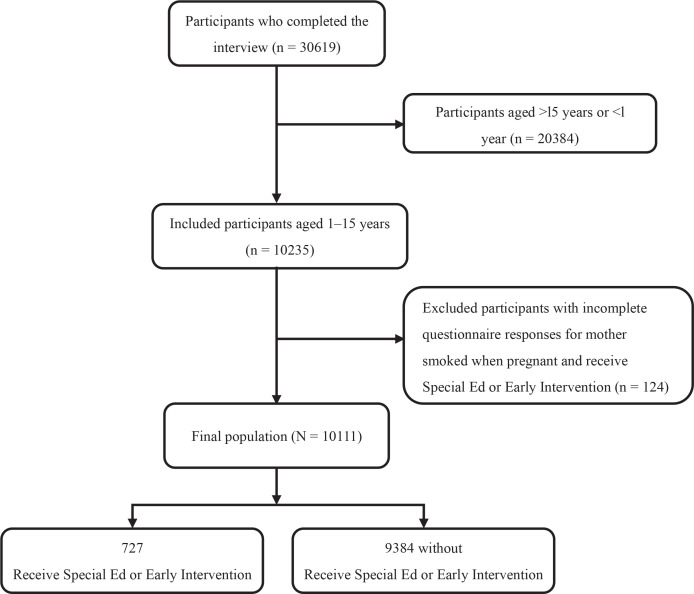
Study flow diagram of individuals aged 1–15 years who participated in the survey NHANES, 2003–2008 (N=10111)

For the purposes of this study, the focus was on individuals aged 1–15 years who participated in the survey. The criteria for inclusion were based on the availability of responses to the survey. The exclusion criteria were for individuals who lacked information about maternal smoking during pregnancy and for individuals who lacked information about access to special education or early intervention services (Figure 1).

### Maternal smoking during pregnancy

In the NHANES 2003–2008 dataset, the variable ‘Mother smoked when pregnant’ was assessed among participants aged 1–15 years through a questionnaire asking if their biological mother smoked at any time during her pregnancy with the participant. Responses affirming maternal smoking were classified as ‘maternal smoking during pregnancy’, while negative responses were classified as ‘no maternal smoking during pregnancy’.

### Receipt of special education or early intervention

For the assessment of special education or early intervention services, children and adolescents aged 1–15 years were selected within the same dataset. Physical functioning was determined using a questionnaire that inquired whether the participants received special education or early intervention services. Those who confirmed that they did, were categorized as ‘receiving special education or early intervention’, and those who did not were categorized as ‘not receiving special education or early intervention’.

### Covariates

This study examined a range of potential covariates: participant’s age, gender, race, household education level, poverty income ratio (PIR), household size, health insurance status^20^, mother’s age at the time of child’s birth, birth weight, and newborn care at a health facility^21^. Race was categorized as Non-Hispanic White, Non-Hispanic Black, Mexican American, or Other^22^. Household education level was classified as <9 years, 9–12 years, or >12 years of education^23^. Family income was categorized based on PIR as low (≤1.3), medium (1.31–3.5), or high (>3.5)^24,25^. Household size was coded as ≤4, or >4^23^. Mother’s age at child’s birth was classified into three categories: <25 years, 25–35 years, or >35 years^26^. Birth weight was categorized as <2500 g or ≥2500 g^21^.

Missing data for covariates such as household education level (3.1% missing), PIR (5.5% missing), health insurance status (0.5% missing), mother’s age at child’s birth (0.7% missing), and birth weight (2.4% missing) were addressed using multiple imputation with a fully conditional specification (FCS) method^27^.

### Statistical analysis

This research was a secondary analysis of publicly available datasets. Categorical variables were presented as frequencies and percentages (%), while continuous variables were described either as mean and standard deviation (SD) or median and interquartile range (IQR), contingent on their distribution. For data with a normal distribution, we evaluated group differences with one-way analysis of variance, for skewed data we employed the Kruskal-Wallis test, and for categorical data we used the chi-squared test. We ascertained the association between maternal smoking during pregnancy and need for early intervention or special education, using logistic regression to evaluate odds ratios (ORs) and 95% confidence intervals (CIs).

Three models were developed for multivariable logistic regression analysis. Model 1: adjusted for age, gender, race/ethnicity, household education level, poverty income ratio. Model 2: as for Model 1 plus household size and health insurance status. Model 3: as for Model 2 plus mother’s age at the child’s birth, birth weight, and newborn care at health facility.

We also investigated potential modifiers of the relationship between maternal smoking and the need for special education or early intervention services, including sex, age (divided into <6 years and ≥6 years), health insurance status, poverty income ratio, birth weight, and newborn care at health facility. Heterogeneity among subgroups was ascertained using multivariable logistic regression, and we explored interactions between subgroups and maternal smoking using likelihood ratio tests.

In the sensitivity analysis, participants with missing covariate data were excluded to verify the consistency of the observed trends with those derived from multiple imputations. Given the use of existing data sets, no prior statistical power calculations were conducted. We used the R statistical software (http://www.R-project.org, The R Foundation) and Free Statistics software version 1.9, for all the analyses. Descriptive statistics were compiled for all participants, with two-tailed p<0.05 considered statistically significant.

## RESULTS

### Study population

A total of 30619 participants completed the interview in the NHANES between 2003 and 2008. Of these, 20384 were excluded because they were either >15 years or <1 year. An additional 124 participants were excluded due to incomplete questionnaire responses regarding maternal smoking during pregnancy and receipt of special education or early intervention services. Consequently, this cross-sectional analysis analyzed data from 10111 participants. The detailed inclusion and exclusion criteria are shown in Figure 1.

### Baseline characteristics

Table 1 presents the baseline characteristics of participants stratified by maternal smoking status during pregnancy. Out of the selected individuals, 727 (7.2%) received special education or early intervention services. A total of 1504 participants (14.9%) were exposed to maternal smoking during pregnancy. The median age of the participants was 8 years (IQR: 3.0–12.0), and 5071 (50.2%) were male. The data showed that mothers who smoked during pregnancy were more likely to be Non-Hispanic White, uninsured, have a lower family income, and belong to households with a higher education level. Additionally, these mothers were more likely to give birth to newborns with lower birth weight who required care at a health facility and necessitated special education or early intervention services.

**Table 1:** Baseline characteristics of selected participants of prenatal smoking and no smoking among mothers whose children were aged 1–15 years, NHANES, 2003–2008 (N=10111)

*Characteristics*	*All* *n (%)*	*No smoking* *n (%)*	*Smoking* *n (%)*	*p*
**Total**	10111	8607	1504	
**Age** (years), median (IQR)	8.0 (3.0–12.0)	7.0 (3.0–12.0)	8.0 (4.0–12.0)	0.012
**Gender**				0.271
Male	5071 (50.2)	4297 (49.9)	774 (51.5)	
Female	5040 (49.8)	4310 (50.1)	730 (48.5)	
**Race/ethnicity**				<0.001
Non-Hispanic White	2894 (28.6)	2129 (24.7)	765 (50.9)	
Non-Hispanic Black	2940 (29.1)	2539 (29.5)	401 (26.7)	
Mexican American	3078 (30.4)	2894 (33.6)	184 (12.2)	
Other	1199 (11.9)	1045 (12.1)	154 (10.2)	
**Household education level** (years)				<0.001
<9	1209 (12.0)	1133 (13.2)	76 (5.1)	
9–12	2078 (20.6)	1695 (19.7)	383 (25.5)	
>12	6824 (67.5)	5779 (67.1)	1045 (69.5)	
**Poverty income ratio**				<0.001
Low (≤1.30)	4527 (44.8)	3745 (43.5)	782 (52)	
Medium (1.31–3.50)	3630 (35.9)	3103 (36.1)	527 (35)	
High (>3.50)	1954 (19.3)	1759 (20.4)	195 (13)	
**Household size**				0.061
≤4	7383 (73.0)	6255 (72.7)	1128 (75)	
>4	2728 (27.0)	2352 (27.3)	376 (25)	
**Health insurance status**				<0.001
Not insured	1381 (13.7)	1228 (14.3)	153 (10.2)	
Insured	8730 (86.3)	7379 (85.7)	1351 (89.8)	
**Mother’s age at child’s birth** (years), mean ± SD	26.0 ± 6.1	26.1 ± 6.1	25.8 ± 6.3	0.092
**Birth weight** (g), mean ± SD	3080.7 ± 637.5	3105.1 ± 630.0	2941.1 ± 662.0	<0.001
**Newborn care at health facility**				<0.001
No	8783 (86.9)	7531 (87.5)	1252 (83.2)	
Yes	1328 (13.1)	1076 (12.5)	252 (16.8)	
**Special education**				<0.001
No	9384 (92.8)	8065 (93.7)	1319 (87.7)	
Yes	727 (7.2)	542 (6.3)	185 (12.3)	

IQR: interquartile range.

### Relationship between maternal smoking during pregnancy and receipt of special education or early intervention

**Figure 2:**
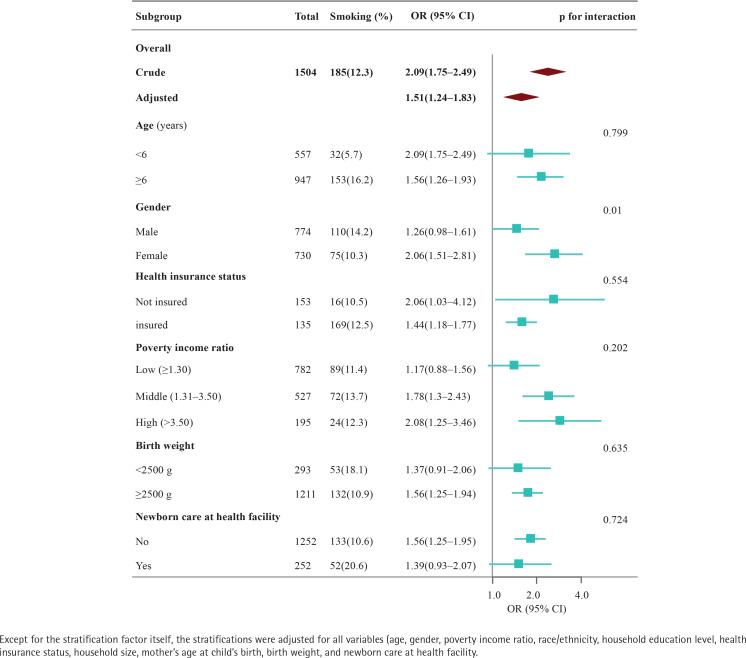
Association between maternal smoking during pregnancy and receipt of special education or early intervention according to the general characteristics

Univariate analysis revealed significant associations between receiving special education or early intervention and the factors: age, gender, race, household education level, PIR, health insurance status, birth weight, care at a health facility, and maternal smoking during pregnancy (Table 2). After adjusting for potential confounders, maternal smoking during pregnancy remained positively associated with the receipt of special education or early intervention (AOR=1.51; 95% CI: 1.24–1.83, p<0.001) (Table 3 and Figure 2).

**Table 2:** The association of covariates and receipt of special education or early intervention among children and adolescents aged 1–15 years, NHANES, 2003–2008 (N=10111)

*Variable*	*OR (95% CI)*	*p*
**Age** (years)	1.11 (1.09–1.13)	<0.001
**Gender**		
Male ®	1	
Female	0.47 (0.40–0.56)	<0.001
**Race/ethnicity**		
Non-Hispanic White ®	1	
Non-Hispanic Black	1.02 (0.85–1.22)	0.856
Mexican American	0.51 (0.41–0.63)	<0.001
Other	0.62 (0.47–0.82)	0.001
**Household education level** (years)		
<9 ®	1	
9–12	2.03 (1.46–2.81)	<0.001
>12	1.93 (1.43–2.60)	<0.001
**Poverty income ratio**		
Low (≤1.30) ®	1	
Medium (1.31–3.50)	1.02 (0.86–1.20)	0.823
High (>3.50)	0.78 (0.62–0.97)	0.024
**Household size**		
≤4 ®	1	
>4	1.08 (0.91–1.27)	0.393
**Health insurance status**		
Not insured ®	1	
Insured	1.55 (1.20–2.00)	0.001
**Mother’s age at child’s birth** (years)		
<25 ®	1	
25–34	0.87 (0.74–1.01)	0.072
≥35	0.99 (0.74–1.33)	0.973
**Birth weight** (g)		
<2500 ®	1	
≥2500	0.51 (0.42–0.61)	<0.001
**Newborn care at health facility**		
No ®	1	
Yes	2.32 (1.94–2.78)	<0.001
**Mother smoked while pregnant**		
No ®	1	
Yes	2.09 (1.75–2.49)	<0.001

Group differences were evaluated using one-way analyses of variance (for normally distributed data), Kruskal-Wallis tests (for skewed data), and chi-squared tests (for categorical data) (p<0.05). ® Reference categories.

**Table 3:** Association between maternal smoking status during pregnancy and receipt of special education or early intervention among children and adolescents aged 1–15 years, NHANES, 2003–2008 (N=10111)

*Variable*	*Total* *n*	*Model 1* *AOR (95% CI)*	*p*	*Model 2* *AOR (95% CI)*	*p*	*Model 3* *AOR (95% CI)*	*p*
No smoking ®	8607	1		1		1	
Smoking	1504	1.67 (1.38–2.01)	<0.001	1.65 (1.37–1.99)	<0.001	1.51 (1.24–1.83)	<0.001

AOR: adjusted odds ratio. Model 1: adjusted for age, gender, poverty income ratio, race/ethnicity, and household education level. Model 2: adjusted as for Model 1 plus household size, health insurance status. Model 3: adjusted as for Model 2 plus mother’s age at child’s birth, birth weight, newborn care at health facility. ® Reference category.

### Stratified analyses based on additional variables and sensitivity analysis

In order to elucidate the nuances within our findings, we further conducted subgroup analyses. Overall, among the 1504 participants identified with maternal smoking during pregnancy, 12.3% were reported to have received special education or early intervention. The crude odds ratio (OR) for receiving these services was 2.09 (95% CI: 1.75–2.49), which adjusted to 1.51 (95% CI: 1.24–1.83) after controlling for the same confounders.

Age-specific analysis showed that for participants aged ≥6 years, the AOR was 1.56 (95% CI: 1.26–1.93), indicating a statistically significant association with receiving special education or early intervention. This association was not significant in children aged <6 years (OR=1.4; 95% CI: 0.89–2.21).

Gender stratification revealed a statistically significant association for females (AOR=2.06; 95% CI: 1.51–2.81) compared to males who did not show a significant association (AOR=1.26; 95% CI: 0.98–1.61).

Considering health insurance status, non-insured individuals had an OR of 2.06 (95% CI: 1.03–4.12), while insured individuals had an OR of 1.44 (95% CI: 1.18–1.77), both indicating significant associations.

Analysis by family income showed that individuals from middle-income families had an OR of 1.78 (95% CI: 1.3–2.43), and those from high-income families had an OR of 2.08 (95% CI: 1.25–3.46), suggesting a stronger association in these groups compared to those from low-income families, which was not statistically significant (OR=1.17; 95% CI: 0.88–1.56).

Birth weight subgroup analysis indicated a significant association for children with a birth weight of ≥2500 g (OR=1.56; 95% CI: 1.25–1.94), whereas the association was not significant for those with a birth weight <2500 g (OR=1.37; 95% CI: 0.91–2.06).

Finally, whether newborn care was received at a health facility did not significantly affect the association with maternal smoking, with OR of 1.56 (95% CI: 1.25–1.95) for those who did not receive care and 1.39 (95% CI: 0.93–2.07) for those who did.

The p-values for interaction suggest that the effect of maternal smoking on receiving special education or early intervention did not significantly vary by age, health insurance status, poverty income ratio, birth weight, or newborn care at a health facility, with the exception of gender (p=0.01), indicating a possible interaction effect. However, due to the potential for multiple tests and the similar directionality of associations, the clinical significance of this finding may be limited. Sensitivity analyses, which excluded individuals with missing covariates, yielded similar results after adjusting for multivariable logistic analyses (Supplementary file Table S1).

## DISCUSSION

This cross-sectional study identified a positive association between maternal smoking during pregnancy and the need for special education or early intervention services in American children and adolescents. Sensitivity analyses confirmed a robust association between these variables. The percentage of participants that were exposed to maternal smoking during pregnancy, was consistent with previous studies in the US^28^.

Previous research has suggested that maternal smoking during pregnancy can increase the likelihood of developmental disabilities. For instance, a Shanghai-based cross-sectional study of 8586 children aged 3–6 years found that those exposed to maternal active smoking *in utero* had a higher risk of developmental coordination disorder compared to those unexposed^29^. Similarly, Minatoya et al.^[Bibr CIT0013]13^ observed an increased risk of difficulties related to behavior and hyperactivity/inattention at pre-school age in children of mothers who smoked during pregnancy. The Raine Study noted increased rates of conduct disorder symptoms at the age of 14 years in the offspring of smoking mothers^30^. However, few studies have assessed the impact of maternal smoking on developmental disorders, as indicated by the use of special education services or early intervention in the US population.

The present study contributes to the literature by demonstrating that children whose mothers smoked during pregnancy were more likely to require special education or early intervention services (AOR=1.51; 95% CI: 1.24–1.83) after accounting for variables such as age, gender, poverty income ratio, race/ethnicity, household education level, household size, health insurance status, mother’s age at child’s birth, birth weight, and newborn care at a health facility.

Although the precise molecular mechanisms by which maternal smoking during pregnancy contributes to developmental disabilities remain elusive, our findings are consistent with existing evidence. Epigenetic modifications, such as DNA methylation, have been proposed as a mechanism by which environmental factors influence human disease. Prior research has linked maternal smoking during pregnancy to changes in placental DNA methylation and RNA expression, as well as DNA methylation in offspring^31-33^. Studies have also investigated the effects of maternal smoking on epigenetic alterations in the human brain. One prospective study used magnetic resonance imaging to assess brain morphology in children aged 6–8 years, revealing that prenatal tobacco exposure was associated with reduced brain volumes, including smaller cortical gray and white matter volumes, and regional cortical thinning in the superior frontal, superior parietal, lateral occipital, and precentral cortex^34^. Furthermore, Semick et al.^[Bibr CIT0035]35^ analyzed RNA sequencing data from post-mortem fetal human prefrontal cortex tissue, identifying 12 genes with differential expression, which underscores the impact of maternal smoking on the developmental trajectories of the prenatal human brain. Another study examining fetuses that were aborted for non-medical reasons, revealed that *in utero* smoking exposure altered patterns of DNA methylation and gene expression, correlating with a reduction in mature neurons, possibly driven by nicotine exposure^36^. Collectively, these studies provide molecular insights suggesting that maternal smoking during pregnancy can disrupt neurodevelopmental pathways and potentially elevate the risk of neuropsychiatric disorders in offspring.

### Limitations

This study has several limitations. Firstly, the assessment of maternal smoking during pregnancy relied on a single self-reported question rather than more precise measures, such as the duration and quantity of smoking. Secondly, despite adjustments for numerous confounders, potential biases may remain due to unaccounted factors like alcohol consumption during pregnancy and maternal psychopathology^12^, which warrant further investigation. Thirdly, the impact of non-random missing data cannot be dismissed, given the baseline differences between included and excluded participants. In addition, outreach to non-US populations has been limited.

Lastly, the cross-sectional design precludes causal inferences. Prospective cohort studies are needed to elucidate the causal relationships between maternal smoking during pregnancy and the requirement for special education or early intervention services.

## CONCLUSIONS

This observational study suggested an association between maternal smoking during pregnancy and an increased need for special education or early intervention services in offspring. The data, derived from the NHANES 2003–2008, indicate this association persists even after adjusting for various sociodemographic factors. However, due to the limitations inherent in the study’s design, longitudinal studies are necessary to explore the causative mechanisms and to consider additional confounding variables that were not included in this study. Despite these limitations, the findings underscore the importance of public health interventions aimed at reducing smoking during pregnancy to potentially diminish the risk of developmental disabilities in children.

## Supplementary Material



## Data Availability

The data supporting this research are available from the following source: http://www.cdc.gov/nchs/nhanes.htm
