# Supplementary material for: Association between maternal smoking during pregnancy and developmental disabilities in US children and adolescents: A cross-sectional study from NHANES
Source: Tob Induc Dis. 2025 Feb 10;23:10.18332/tid/200339. doi: 10.18332/tid/200339 (PMC11808298; doi:10.18332/tid/200339)
Supplement: Supplementary file 1 [file TID-23-13-s1.pdf]

Supplemental files

S1 Association between maternal smoking status during pregnancy niacin and receive special E-d or early Intervention among children and adolescents aged 1–15 years, NHANES, 2003-2008 (N=10111)（Excluding missing covariates sensitivity analyses）

	OR（95% CI）						
Variable	No.	Model 1	p-value	Model 2	p-value	Model 3	p-value
No smoking	8438	1(Ref)		1(Ref)		1(Ref)	
Smoking	644	1.67 (1.36~2.04)	<0.001	1.66(1.36~2.04)	<0.001	1.53(1.24~1.88)	<0.001

*Missing data for covariates such as household education level (3.1% missing), PIR (5.5% missing), health insurance status (0.5% missing), mother’s age at child’s birth (0.7% missing), and birth weight (2.4% missing)

© 2025 Miao H. et al.
